# SUP: a probabilistic framework to propagate genome sequence uncertainty, with applications

**DOI:** 10.1093/nargab/lqad038

**Published:** 2023-04-24

**Authors:** Devan Becker, David Champredon, Connor Chato, Gopi Gugan, Art Poon

**Affiliations:** Department of Pathology and Laboratory Medicine, Schulich School of Medicine and Dentistry, Western University, London, Ontario, Canada; Public Health Agency of Canada, National Microbiology Laboratory, Public Health Risk Sciences Division, Guelph, Ontario, Canada; Department of Pathology and Laboratory Medicine, Schulich School of Medicine and Dentistry, Western University, London, Ontario, Canada; Department of Pathology and Laboratory Medicine, Schulich School of Medicine and Dentistry, Western University, London, Ontario, Canada; Department of Pathology and Laboratory Medicine, Schulich School of Medicine and Dentistry, Western University, London, Ontario, Canada

## Abstract

Genetic sequencing is subject to many different types of errors, but most analyses treat the resultant sequences as if they are known without error. Next generation sequencing methods rely on significantly larger numbers of reads than previous sequencing methods in exchange for a loss of accuracy in each individual read. Still, the coverage of such machines is imperfect and leaves uncertainty in many of the base calls. In this work, we demonstrate that the uncertainty in sequencing techniques will affect downstream analysis and propose a straightforward method to propagate the uncertainty. Our method (which we have dubbed Sequence Uncertainty Propagation, or SUP) uses a probabilistic matrix representation of individual sequences which incorporates base quality scores as a measure of uncertainty that naturally lead to resampling and replication as a framework for uncertainty propagation. With the matrix representation, resampling possible base calls according to quality scores provides a bootstrap- or prior distribution-like first step towards genetic analysis. Analyses based on these re-sampled sequences will include a more complete evaluation of the error involved in such analyses. We demonstrate our resampling method on SARS-CoV-2 data. The resampling procedures add a linear computational cost to the analyses, but the large impact on the variance in downstream estimates makes it clear that ignoring this uncertainty may lead to overly confident conclusions. We show that SARS-CoV-2 lineage designations via Pangolin are much less certain than the bootstrap support reported by Pangolin would imply and the clock rate estimates for SARS-CoV-2 are much more variable than reported.

## INTRODUCTION

Generating a genetic sequence from a biological sample is a complex process. Nucleic acids must be extracted from the sample while avoiding contamination by foreign material. If working with RNA, then we must use a reverse transcriptase reaction (which has a high base misincorporation rate) to convert the RNA into DNA. Polymerase chain reaction (PCR) amplification is often employed to enrich the sample for the target of interest. For next-generation sequencing (NGS) protocols, we have to generate a sequencing library, for instance by random shearing of nucleic acids into fragments that are ligated onto special ‘adaptors’. NGS procedures such as sequencing by synthesis suffer from greater error rate relative to conventional Sanger dye-terminator sequencing, although these rates have continued to improve with new technologies (1–3). In addition, the short reads produced by NGS platforms need to be aligned—either by alignment against a reference genome, *de novo* assembly, or a combination of the two—to reconstruct a consensus sequence using one or more bioinformatic programs. Errors can be introduced in any one of these steps (4,5).

In some cases, naturally occurring variation, i.e. genetic polymorphisms, or variation induced by experimental error is directly quantified and encoded into the output. For example, mixed peaks in sequence chromatograms produced from dye-terminator sequencing by capillary electrophoresis are assigned standard IUPAC codes (e.g. Y for C or T) when the base calling program cannot determine which base is dominant (6). Ewing and Green (7) and Richterich (8) argued that estimates of the base call quality, quantified as Phred quality scores, can be an accurate estimate of the number of errors that the machines at the time would make, but improvements to these error probabilities have been proposed (9,10). Nevertheless, Phred scores remain the standard means of reporting the estimated error probabilities for current sequencing platforms. Generally, these scores are either used to censor the base calls (i.e. label them ‘N’ rather than A, T, C or G) if the estimated probability of error exceeds a predefined threshold or remove the sequence from further analysis if the total number of censored bases exceeds a maximum tolerance (e.g. (5,11,12)). Some authors/tools use more sophisticated models, such as Wu *et al.* (13) who use statistical models that incorporate read depth to determine a probability of a sequencing error, but still use the resultant reads to form a consensus sequence with no measure of uncertainty. Furthermore, some studies have extended the concept of per-base error probabilities to calculate the joint likelihoods of partial or full sequences. For example, DePristo *et al.* (14) and Gompert and Buerkle (15) incorporate adjusted Phred scores into a likelihood framework to generate more accurate estimates of genetic diversity within a population; this approach has subsequently been used to develop new estimators of genetic diversity (16). Kuo *et al.* (17) recently used a similar approach to develop a statistical test of whether a given genome sequence is consistent with a specified alternative sequence. In general, the reported error probabilities from NGS technologies are primarily used for filtering low quality sequences and improving alignment algorithms (which both result in a consensus sequence that is assumed to be error-free) or for hypothesis tests concerning small collections (usually pairs) of sequences.

The uncertainty present in the sequences are most often ignored entirely. For example, methods for sequence alignment and homology searches generally employ heuristic algorithms that utilize similarity scores that do not explicitly incorporate the probabilities of sequencing errors. The problem of unacknowledged uncertainty is exacerbated when each sequence represents the consensus of diverse copies of a genome, such as rapidly evolving virus populations where genuine polymorphisms are confounded with sequencing error. See (18) for more criticisms of the use of consensus sequences, along with visualizations ((19), called *sequence logos*) to display the deviations from a consensus.

Though rare, some studies have proposed methods for propagation of uncertainty from one step to later steps of an analysis. O’Rawe *et al.* (5) suggest methods for propagation of sequence-level uncertainty into determining whether two subjects have the same alleles, as well as estimating confidence intervals for allele frequencies. Another exception can be found in (20), who incorporate an assumed or estimated error rate for the entire sequence into the calculation of a phylogenetic tree and found that incorporation of errors makes the inferred branch lengths much closer to the true (simulated) branch lengths. Though they did not use nucleotide-level uncertainty, Gompert and Buerkle (15) incorporate the coverage of NGS technologies as part of the uncertainty of estimates for the frequency of alleles in a population. Clement *et al.* (21) present an alignment algorithm (called GNUMAP) that takes nucleotide-level uncertainty into account. Their method incorporates Position Weight Matrices into a method of scoring multiple possible matches against a reference genome in order to choose the best alignment. These studies are the exceptions, rather than the rules, and their methods have not yet attained widespread use.

We present a simple general-purpose framework that can be incorporated into any analysis of genetic sequence data. This framework involves converting the uncertainty scores into a matrix of probabilities, and repeatedly sampling from this matrix and using the resultant samples in downstream analysis. Unlike likelihood-based approaches, we do not make assumptions about the underlying patterns or distributions in the data. In so doing, we can gain more accurate estimation of the errors at the expense of computation time. Our technique is amenable to quality score adjustments prior to applying our methods. We demonstrate the impact of propagating sequence uncertainty by applying our methods to the problem of classifying SARS-CoV-2 genomes into predefined clusters known as ‘lineages’ (22), several of which correspond to variants carrying mutations that are known to confer an advantage to virus transmission or infectivity. We also analyse a collection of SARS-CoV-2 sequences to demonstrate that the estimated rate of new mutations is much more variable than studies relying on deterministic sequences would conclude.

## MATERIALS AND METHODS

### Probabilistic representation of sequences

Here, we describe two theoretical frameworks to model sequence uncertainty at the *nucleotide level* or at the *sequence level*. In both frameworks, the sequence of nucleotides from a biological sample is not treated as a single unambiguous observation (known without error), but rather as a collection of possible sequences weighted by their probability.

#### Nucleotide-level uncertainty

To represent the uncertainty at each position along the genome we introduce the following matrix, which we will refer to as a probabilistic sequence and denote as $\mathcal {S}$:

(1)

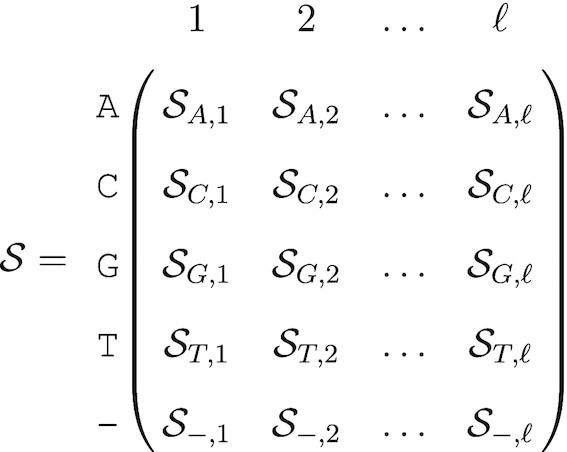

Each column represents a position in a nucleotide sequence of length ℓ. Each row represents one of the four nucleotides A,C,G,T, as well as an empty position ‘–’ that symbolizes a recorded deletion rather than missing data. Hence, $\mathcal {S}$ is a 5 × ℓ matrix.

The elements of the probability sequence represent the probability that a nucleotide exists at a given position, with a special case for the empty position –:

(2)

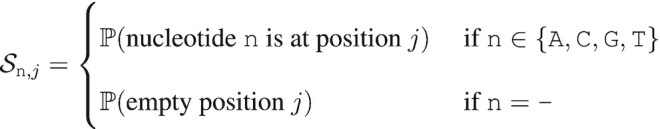

Note that we have for all 1 ≤ *j* ≤ ℓ:


(3) $$\begin{equation*} \sum _{n} \mathcal {S}_{n, j} = 1 \end{equation*}$$

Also, the sequence length is stochastic if $0<\mathcal {S}_{\texttt {-},i}<1$ for at least one *i*. The nucleotide (or deletion) drawn at each position is independent from all the others, so there are up to 5^ℓ^ possible different sequences for a given probabilistic nucleotide sequence, but these sequences are *not* equally probable.

A major limitation of this probabilistic representation of a sequence is that we lose all information on linkage disequilibrium. This is especially problematic for recording insertions because insertions with *L* ≥ 2 nucleotides are treated as *L* independent single nucleotide insertions. Instead, we assume that every nucleotide is an independent observation. For example, a probability sequence populated from short read data from a diverse population would not store the information that two polymorphisms were always observed in the same reads, i.e. in complete linkage disequilibrium. We also lose information about autocorrelation in sequencing error, such as clusters of miscalled bases associated with later cycles of sequencing-by-synthesis platforms. Sequence chromatograms and base quality scores are affected by the same loss of information.

We note that this representation is similar to the ‘CATG’ file type as described in (23), which indicates the likelihoods of each nucleotide in an aligned mapping for multiple taxa. This file type is able to be used by RAxML-NG to estimate an overall error rate which is then used to estimate phylogenetic trees. A reviewer has pointed out that the bio++ library contains parsers for a probabilistic version of the FASTA format, called PASTA. We have not found documentation for this format, but are hopeful that our methods promote greater use of probabilistic formats like this. Our probability sequence is also similar in concept to Position Weight Matrices (PWMs, (24)) which are built according to the frequency of each base at each position of a multiple alignment. Our construction differs in that we are creating one matrix per sequence where the entries are weighted according to error probability within that sequence, rather than one matrix for a collection of sequences. However, methods that accept PWMs will be applicable to our probability sequences (and *vice-versa*).

It is also possible to determine the sequence-level uncertainty as the product of nucleotide uncertainties for all possible sequences. This could be useful for creating an ordered list of the most likely sequences or removing any sequences that are not biologically plausible (e.g. sequences missing a crucial amino acid, especially a start or stop codon). A full discussion of this is in the supplementary materials.

#### Sequence-level uncertainty

A significant problem of storing probabilities at the level of individual nucleotides is that generating a sequence from this matrix requires drawing ℓ independent outcomes. For example, the reference SARS-CoV-2 genome is 29 903 nucleotides, and a substantial number of naturally-occurring sequence insertions have been described. Thus, it would not be surprising if ℓ exceeded 30 000 nucleotides (nt). The majority of these technically possible 5^ℓ^ sequences are not biologically plausible. Therefore, we formulate an ordered subset $\mathcal {B}= (\mathcal {B}_i)_{i\in \lbrace 1\ldots m\rbrace }$ of the first *m* most likely sequences, which are ranked in descending order by the joint probability of nucleotide composition. Note that the sequences in $\mathcal {B}$, $\mathcal {B}_i$, do not necessarily have the same length. The observed genetic sequence, s*, is a sample from a specified discrete probability distribution *a*:


(4) $$\begin{equation*} \mathbb {P}(s^* = \mathcal {B}_i | i ... m) = a(i) \end{equation*}$$

This compact and approximate representation drastically reduces the number of operations to one sample, after some pre-processing to calculate *a*. The observed plurality sequence s* (the sequence consisting of the most likely base at each position) is guaranteed to be a member of $\mathcal {B}$ if $\mathcal {S}_{s(j), j} >0.5\;\forall \;j$ where *s*(*j*) is the *j*-th nucleotide of s*; indeed, it is guaranteed to be the highest ranked member *i* = 0. We refer to any member of the set $\mathcal {B}$ as a *sequence-level probabilistic sequence*. Note that because *a* is a probability distribution, we must have $\sum _{i=1}^m a(i) = 1$. In other words, this probability is conditional on the sequence being in $\mathcal {B}$.

For example, suppose that we have the following nucleotide-level probabilistic sequence:

(5)

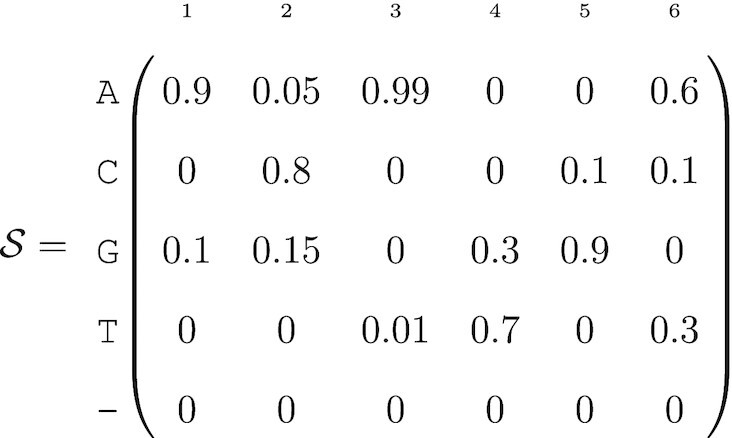

such that there are 2 × 3 × 2^3^ × 3 = 144 possible sequences. The most likely sequence has the highest joint nucleotide probability: ACATGA with probability 0.2694 (0.9 × 0.8 × 0.99 × 0.7 × 0.9 × 0.6). If there is a positive probability of deletion for at least one position, then the sequence has a variable length. Large genomes or sequencing targets will result in vanishingly small probabilities for all sequences, and thus calculations on the log scale may be necessary to reduce the chance of numerical underflow.

Table 1 demonstrates the calculation of sequence-level uncertainties using the values in (5). The probability column is the product of the matrix entries for each nucleotide. If the four sequences shown are the only biologically plausible sequences, then the normalized probabilities can be expressed as *a*(*i*).

**Table 1.** Biologically plausible sequences with probabilities defined by (5)

Sequence	Probability	*a*(*i*)
$\mathcal {B}_1$ = ACATGA	0.299	*a*(1) = 0.467
$\mathcal {B}_2$ = ACATGT	0.150	*a*(2) = 0.233
$\mathcal {B}_3$ = ACAGGA	0.128	*a*(3) = 0.200
$\mathcal {B}_4$ = ACAGGT	0.064	*a*(4) = 0.100

In summary, sequence-level probabilistic sequences offer a convenient way to define a (much) smaller set of possible sequences than the potential 5^ℓ^ nucleotide-level probabilistic sequences. This set will be used to generate sequences randomly for downstream analyses. The size of this set (noted *m* above) is arbitrarily determined by users.

### Constructing the probability sequence

In most next-generation sequencing applications, the estimated probability of sequencing error is quantified with the quality (or ‘Phred’) score attributed to each base call produced by sequencing instrument. The quality score *Q* is directly related to this estimated error probability: ε = 10^−*Q*/10^ (7), where *Q* typically ranges between 1 and 60 (with 60 being the lowest probability of error), depending on the sequencing platform and version of base-calling software. It is important to note that this quality score only measures the probability of error from the machine; 1 − ε is an estimate of the probability of no sequencing errors and does not account for any other source of error.

More formally, the probability that the base call is correct is expressed as:


(6) $$\begin{equation*} \mathbb {P}(\text{nucleotide}=X \, \, |\, \, \text{observed nucleotide} = X) = 1 - \epsilon \end{equation*}$$

Unfortunately, quality scores have no information on the probabilities of the three other possible nucleotides if the base call is incorrect. In the absence of information about the other bases (such as with consensus-level FASTQ or FASTA files), we assume that these other probabilities are uniformly distributed.

Raw short read data are typically recorded in a FASTQ format that stores both the sequences (base calls) and base-specific quality scores for each short read. Since the reads often correspond to different positions of the target nucleic acid, e.g. randomly sheared genomic DNA, it is necessary to align the reads to identify base calls on different reads that represent the same genome position. This alignment step can be accomplished by mapping reads to a reference genome, by the *de novo* assembly of reads, or a hybrid approach that incorporates both methods. The aligned outputs are frequently recorded in the tabular Sequence Alignment/Map (SAM) format (25). Each row represents a short read, including the raw nucleotide sequence and quality strings; the optimal placement of the read with respect to the reference sequence (as an integer offset); and the compact idiosyncratic gapped alignment report (CIGAR) string, an application-specific serialization of the edit operations required to align the read to the reference. The SAM format contains much more information (https://samtools.github.io/hts-specs/SAMv1.pdf), but for our purposes we only need the placement, sequence, quality, and CIGAR string.

**Figure 1.**
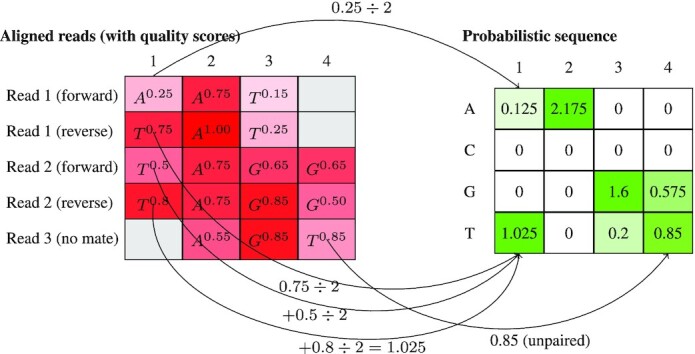
An illustration of constructing a probabilistic sequence from a SAM file. Each row in the matrix on the left is a graphical representation of a short read, and the superscript represents the quality score (from 0 to 1). Half of the quality score from paired end reads is added to the relevant cell in the matrix on the right. In both matrices, the column numbering represents a position on the reference genome. Note that this is an intermediate step prior to ensuring that the columns sum to 1. In the probabilistic sequence, we can see that the consensus sequence would be TAGT, but TAGG is also a very likely sequence given the quality scores.

We employed the following procedure to construct the nucleotide-level probabilistic sequence from the contents of a SAM file. We initialize aligned sequence and quality strings with ‘–’ in all positions before the first read and after the last read, and ‘!’, which corresponds to a quality score of 0 (*Q* = 0), to all other positions. Next, we tokenize the CIGAR string into length-operation tuples, which determine how bases and quality scores from the raw strings are appended to the aligned versions. Deleted bases (‘D’ operations) are not assigned Phred scores, so we assume them to have 0 error probability. The overall process for constructing the probabilistic sequence is demonstrated in Figure 1, including our procedure for including paired-end reads which is explained in a subsequent section. Note that Figure 1 shows an intermediate step prior to column normalization; our algorithm reads the file in one row at a time, which saves on computer memory but means we cannot know the column sums until the process is complete.

### Deletions and insertions

By construction, the nucleotide-level probabilistic sequence would need to be defined with its longest possible length, i.e. a multiple alignment for all reads. Deletions are naturally modelled with our representation but insertions would have to be modelled using deletion probabilities.

(7)

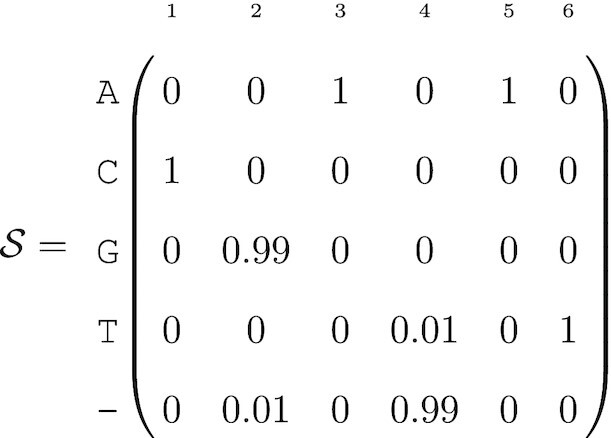

The low deletion probability for position 2 is straightforward to interpret: in about 1% of the reads that contained this position, nucleotide G at position 2 is deleted. The high deletion probability for position 4 means there is a 1% chance of a T insertion at this position (Table 2).

**Table 2.** Sequence-level probabilistic sequence defined by (7)

Sequence	Probability
$\mathcal {B}_1$ = CGAAT	*a*(1) = 0.9799
$\mathcal {B}_2$ = CAAT	*a*(2) = 0.01
$\mathcal {B}_3$ = CGATAT	*a*(3) = 0.01
$\mathcal {B}_4$ = CATAT	*a*(4) = 0.0001

This probability sequence is non-trivial to construct. Consider a short read with two bases inserted at position *j* (say, an A at position *j* + 1 and a T at position *j* + 2) and a short read with one insertion at position *j* (say, a C). It is entirely ambiguous whether the single insertion (C) aligns with the first insertion (A) or the second insertion (T) of the first short read. This is problematic for building up the matrix from reads aligned to the reference sequence. It is conceptually and computationally simpler to start from a populated matrix and sampling insertions. For our purposes, we only consider the pairwise alignment of these sequences with a reference sequence and thus do not consider insertions.

### Paired-end reads

Some NGS platforms (e.g. Illumina) use paired-end reads where the same nucleic acid template is read in both directions. In these situations, we simply adjust all values by a factor of one half. For bases where the paired-end reads overlap, this has the effect of averaging the base probability 1 − ε. For example, if 1 − ε is 90% for Ain one read and 95% Ain its mate, then 0.925 is added to the Arow in $\mathcal {S}^{\prime }$ (with the remaining 0.075 uniformly distributed across the other nucleotides). If the two reads were 70% Aand 55% Cat the same position, then we would increment the corresponding column vector (A, T, C, G) by (0.7/2, 0.1/2, 0.1/2, 0.1/2) for the first read and (0.15/2, 0.15/2, 0.55/2, 0.15/2) for the second, resulting in an addition of (0.425, 0.125, 0.325, 0.125) for this pair. Bases outside of the overlapping region contribute a maximum of 0.5 to $\mathcal {S}^{\prime }$, because the base call on the other read is missing data. This approach has the advantage of making the parsing of SAM files trivially parallelizable since we do not need to know how reads are paired. In addition, the coverage calculated from $\mathcal {S}^{\prime }$ is scaled to the number of templates rather than the number of reads.

### Consensus sequence FASTQ and FASTA files

#### Consensus sequence FASTQ files

Full length or partial genome sequences are now frequently the product of next-generation sequencing, by taking the consensus of the aligned or assembled read data. However, the original read data are often not published alongside the consensus sequence. For example, on 30 September 2022, there were nearly 390 000 SARS-CoV-2 consensus genome sequences available in the Canadian VirusSeq Data Portal. None of the raw NGS data sets associated with these consensus sequences are distributed in this database, however. Less than 6700 (about 1.7%) raw SARS-CoV-2 FASTQ files for samples collected in Canada have been published on the NCBI Sequence Read Archive. On the other hand, some consensus sequences are released in a format where the bases are annotated with quality scores, e.g. FASTQ. There are several programs that provide methods to convert a SAM file into a consensus FASTQ file (9,26,27). These programs use slightly different methods for generating consensus quality scores, but filter quality scores for the majority base. For example, suppose there are three reads with the following base calls at position *j*: A with *Q* = 30, A with *Q* = 31, and C with *Q* = 15. Calculation of the consensus quality score will thereby exclude the *Q* = 15 value and report a quality score calculated from *Q* = 30 and *Q* = 31, with the details of the calculation differing by software.

This omission makes it challenging for us to generate an $\mathcal {S}$ matrix from a consensus FASTQ file. Given the consensus base and its associated quality score at position *j*, we must assume that the other bases are all equally likely with probability ε_*j*_/3 (similar to (17) and Chapter 5 of (23)). For example, let’s assume the output sequence after fragment sequencing and alignment is ACATG and its associated quality scores are respectively *Q* = (60, 30, 50, 10, 40). The probabilistic sequence is:

(8)

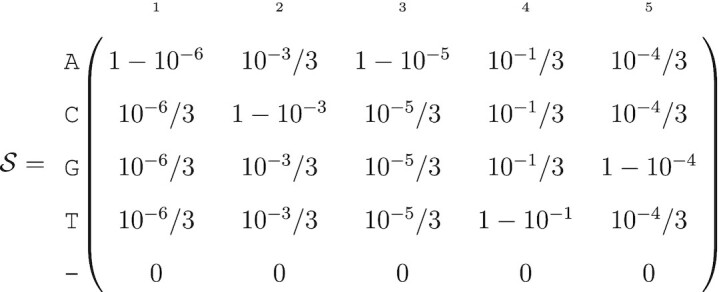

Usually, the genetic sequence ACATG would be considered as certain and quality scores discarded. In contrast, the probability of the sequence ACATG is only 0.899 within the probabilistic sequence framework.

Incorporating deletions in the absence of raw data is also challenging. If one is willing to assume a global deletion rate, then it is possible to extend the parameterization of $\mathcal {S}$. For example, if the probability of a single nucleotide deletion is *d*, then the probability of the called base is (1 − *d*_*j*_)(1 − ε_*j*_) and the other three nucleotides have probability (1 − *d*)ε_*j*_/3. Hence, if we assume the base call is A, the column of the nucleotide-level probabilistic sequence for that position is

(9)

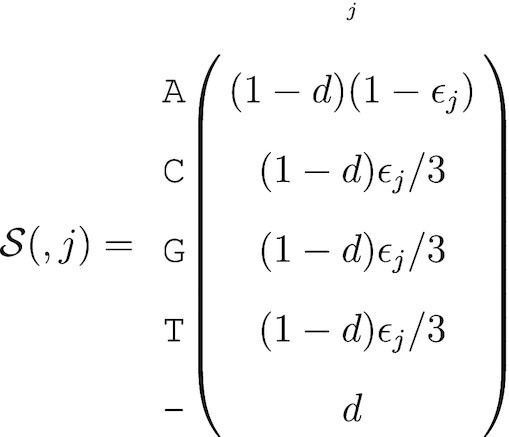

Since the FASTQ file only has a single sequence, we do have the same issues with alignment of differing lengths of insertions. In fact, insertions are only insertions relative to the reference sequence; they can simply be treated as observed nucleotides with an associated quality score. It would be possible to give insertions special treatment, however, by defining a global insertion rate. This insertion rate can be expressed as a deletion rate relative to the observed sequence, and thus one minus the insertion rate can be treated as the deletion rate in the probabilistic sequence. As with the deletion rate, this requires an assumption about a global rate which may be arbitrary.

A primary use of the probability sequence created from these FASTQ files would be to construct a probability sequence as a reference genome for a given category. This would entail collecting all available FASTQ files within a lineage designation and using them in the construction of a probability sequence as if they were short reads in a SAM file, thus creating a lineage-summarising probabilistic sequence. From here, lineage designation for a newly acquired sequence (and its probability sequence) could be performed via comparison of the new sequence with the library of lineage-summarising probabilistic sequences. Such a comparison must properly consider the error structures of the new lineage, which is constructed from short reads and this is fundamentally different from the probabilistic sequences for each lineage, and should be based on the probability of similar consensus sequences rather than similar error structures.

#### Consensus sequence FASTA files

If we do not have access to any base quality information, e.g., the consensus sequence is published as a FASTA file, then our ability to populate $\mathcal {S}$ is severely limited. Any uncertainty that we impose upon the data will be a principled assumption for the purpose of evaluating the robustness of the results to potential or assumed sequence uncertainty. The error probability at the *j* position of the consensus sequence can be simulated as a beta distribution, i.e.,


$$\begin{equation*} \epsilon _j \sim \text{Beta}(\alpha , \beta ) \end{equation*}$$

The called base at position *j* has probability 1 − ε_*j*_, and the remaining bases are assigned ε_*j*_/3. To incorporate deletions, another probability *d* can be generated as the *gap probability*. With these defined, the nucleotide-level probabilistic sequence at the *j*th column (assuming the base call at position *j* was A) can be written as above. This probabilistic sequence is completely fabricated, i.e., not based on any empirical data. However, the sensitivity of an analysis can be evaluated by choosing different values of α, β, and *d* (e.g. based on previous studies) and propagating these uncertainties into downstream analyses. The results from such an analysis would not indicate anything about the sequence itself but could be used to determine how robust the methods are to increased sequence uncertainty.

**Figure 2.**
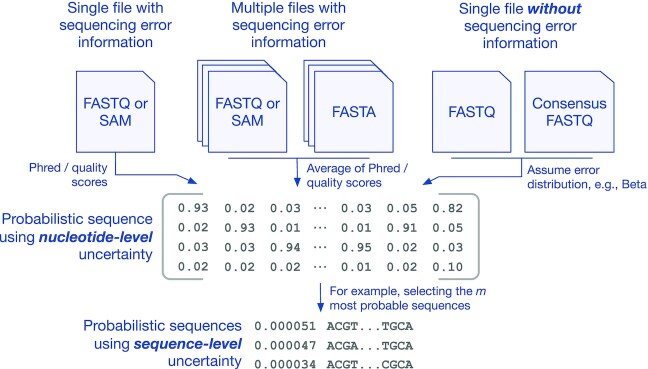
Summary of probabilistic sequences construction. Nucleotide-level probabilistic sequences can be generated from a single FASTQ or SAM file using the sequencing quality information (left). In the case of multiple FASTQ or SAM the user can average the sequencing quality information beforehand (center). When multiple FASTA files are available, the probabilities can be directly informed from the frequencies of nucleotides at each position (center). In the case of a single FASTA file or consensus FASTQ file, the user can assume a probability model for the distribution of sequencing errors (right). Sequence-level probabilistic sequences may be obtained from the nucleotide-level ones, for example by selecting the *n* most probable sequences (bottom).

Figure 2 summarizes the various ways a probabilistic sequence can be obtained depending on the type of data available.

For both the FASTQ and FASTA format, the uniform distribution was chosen for illustrative purposes. We hope that future analyses take uncertainty into account, and each analysis will have unique needs. In the absence of available SAM files, alternate assumptions about the unknown uncertainties can be made. As noted by a reviewer, for viruses such as SARS-CoV-2 it is possible to calculate the per-position frequencies of each letter. In other contexts, there may be other potential assumptions that coincide with known features of the organism.

### Propagation of uncertainty via resampling

The most general way to propagate uncertainty is through resampling. Given $\mathcal {S}$ and assuming that individual nucleotides are independent outcomes we can propagate uncertainty by running downstream analyses on each set of sampled sequences.

At a nucleotide level, we are sampling from a multinomial distribution. If the *j*th column of $\mathcal {S}$ is (0.5, 0.2, 0.2, 0.09, 0.01), then we could sample A with 50% probability, C with 20%, etc. As with other sequence analyses, we can censor the positions that do not have enough coverage. We arbitrarily chose to censor any position that had fewer than 10 reads.

### Implementation

A C program has been written to convert SAM files into our matrix representation. The program assumes that the reads are aligned to a reference, then uses that reference to initiate the matrix. Because of our methods for handling paired reads, the program is able to stream the file line-by-line in a parallel computing environment. However, this C program currently does not output insertions or deletions, and thus they are not part of this algorithm.

The resampling algorithm defined above has been implemented in the R programming language. A shell script is used to repeatedly call the necessary R functions and apply the resampling algorithm to all outputs of the C program until the desired number of samples is obtained. All of the code for this project is available at https://github.com/Poonlab/SUP.

## RESULTS

### SARS-CoV-2 lineage assignment

In this section, we apply the re-sampling method to evaluate the impact of sequencing error on the lineage assignments of SARS-CoV-2. Sequences are sampled from $\mathcal {S}$, assigned a lineage based on the lineage designation algorithm described in (22) using the pangoLEARN tool (Pangolin version 2.3.2, pangoLEARN version 2021-02-21) that the authors have made available (github.com/cov-lineages/Pangolin). This tool uses a decision tree model to determine which lineage a given sequence is most likely to belong to. We demonstrate that even the best available tools are underestimating the variance and therefore producing overconfident conclusions.

#### Data

The data for this application were downloaded from NCBI’s SRA web interface (https://www.ncbi.nlm.nih.gov/sra/?term=txid2697049) on 17 July 2021. Search results were filtered to only include records that had SAM files so that our alignments were consistent with the originating work. We note that the use of pre-aligned SAM files means that we do not have full control over the reference sequence, and thus there may be some difference in the choice of alignment which may lead to probabilistic sequences that are not aligned to each other. In our first application we do only make comparisons within re-samples of a sequence - not between sequences - and our second application involves a multiple sequence alignment in order to find mutations relative to each other. To select which runs to download, an arbitrary selection of 5-10 records from each of 20 non-sequential results pages were chosen. Once collecting the run accession numbers from the search results, an R script was run to download the relevant files and check that all information was complete. Twenty-three out of 275 files were incomplete due to technical errors during the download process and a further four were rejected due to lack of CIGAR strings (the NCBI database automatically converts files uploaded as unaligned FASTQ into the SAM file format without performing alignment), leaving 248 sequences analysed in this work. The SRA accession numbers for the sequences we used are provided in Supplementary Table S1.

#### Re-sampling the probabilistic sequence

Since pangoLEARN is a pre-trained model, assigning lineage designations to a large number of resampled genome sequences is not computationally burdensome. Sampling 5000 different sequences from a probabilistic sequence can be done in a reasonable amount of time, even on a mid-range consumer laptop. Our implementation of the construction of the probabilistic sequence does not output insertions and deletions, so the results in this section are only based on mutations.

**Figure 3.**
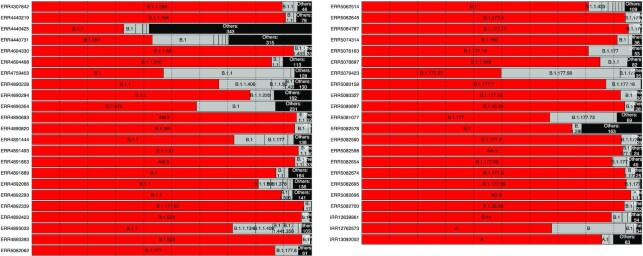
Visualization of called lineages from Pangolin. Red bars indicate the lineage of the most probable sequence and grey bars represent other sequences called from the same SAM file. Any lineage with fewer than 100 observations in the simulated sequences was grouped into the ‘Other’ category. There were 95 sequences total, but we only plotted the ones where the second most common lineage designation had >250 observations.

Figure 3 shows the results of the 49 sequences where there were more than 250 sampled sequences in the second highest lineage call. The consensus sequence is almost always assigned to the same lineage as the majority of the resamples, but the proportion of resamples with the same lineage as the consensus sequence is very rarely 100% and can be as low as 32.86% (accession number ERR4440425). There were 52 cases where the proportion agreeing with the consensus sequence was either exactly 0 or <1%, and these cases occurred when the most common lineage sampled was labelled B.1.1.7 or ‘None’ (sequences are labelled ‘None’ when pangolin’s classification does not reach a confidence threshold). B.1.1.7 represents 6% of our data and is a significantly more infectious lineage that is of special concern to health authorities.

**Figure 4.**
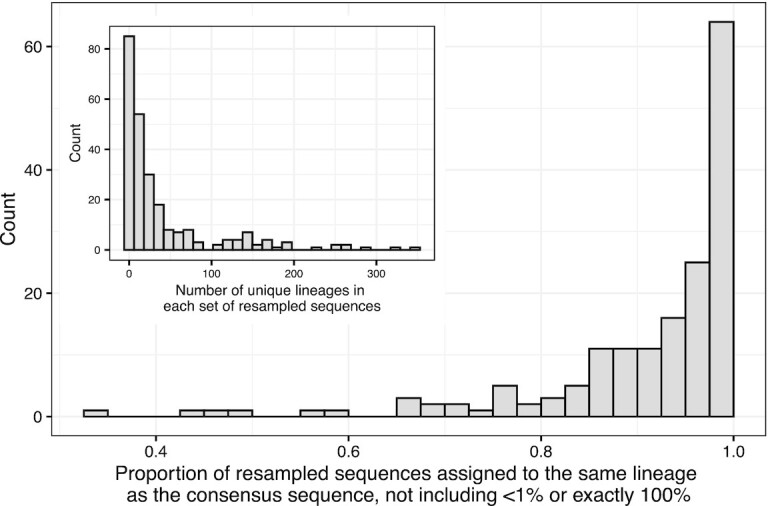
Main plot: Proportion of resampled sequences that are assigned to the same lineage as the consensus sequence. One proportion is calculated for each SAM file. The sets of resampled sequences where the proportion was <1% or exactly 100% are explained in the section titled Re-sampling the probabilistic sequence. Inset: The number of distinct lineage assignments within each set of resampled sequences.

Figure 4 shows both the proportion of lineages assigned to the same lineage as the consensus sequence as well as the number of different lineage assignments for each sequence we analysed. The clear majority of resampled sequences are assigned to the same lineage as the consensus sequence, but there are many cases where the proportion is <80% or even 60%. From the inset, most of the SAM files result in a small number of different lineage assignments, but there are cases where there are more than 100 different alternative lineages that were possible.

### Clock rate estimation for SARS-CoV-2

The molecular clock rate (the number of mutations per site per unit of time) of a phylogenetic tree is found by considering both the number of mutations for each observed sequence relative to the root of the tree and the sample dates of those sequences. Assuming heterochronous sampling dates, the rate of mutations can be estimated by regressing the number of mutations against the sampling date. In the simplest case the clock rate is the slope estimate from a linear regression, thus assuming a fixed clock rate. Polynomial and non-linear clock rates can be estimated (28), as well as Bayesian non-parametric estimates (29).

The clock rate for SARS-CoV-2 is commonly estimated as a fixed rate near 0.001 mutations per site per year (30–34). Using the same resampling methods as above, we estimate a clock rate for trees estimated from each of 50 resamples and for the tree estimated based on the consensus sequences.

To obtain the data, we sampled genomes uniformly from each month of recorded data in GenBank, using filters to ensure that the genomes were complete and had an associated SAM file. We further had to filter out SAM files that were incomplete or did not contain the CIGAR strings necessary for alignment, leaving us with 244 sequences. The associated SRA accession numbers are provided in Supplementary Table S2.

Our re-sampling method will, by definition, introduce other possible mutations beyond what the consensus sequence suggests. Because of this, the apparent number of mutations between a re-sampled genome and the estimated root is a function of the coverage, with more positions read or more uncertainty in the sequence leading to artificially inflated terminal branch lengths. Furthermore, we are sampling nucleotides at each position independently of other positions as well as independently of ancestral sequences. This implies that the estimates of the time for the most recent common ancestor are not reliable. However, assuming that the sequences have comparable levels of uncertainty, each branch increases by a similar amount and the clock rate should not be affected.

The sequences that we acquired did not have comparable levels of uncertainty; the viruses sampled early in the pandemic had considerably higher uncertainty, most likely due to a lack of consistent laboratory guidelines for sequencing this new virus. To account for this, we calculated the sum of $\mathcal {S}^{\prime }$ for each sequence and applied Statistical Process Control techniques to ensure that all of the sequences had a similar level of coverage. In particular, we calculated the mean coverage of the sequences in our data set, $\bar{c}$, and the standard deviation of the coverages, *s*. We removed any sequences outside of $\bar{c} \pm 3 s$, recalculated $\bar{c}$ and *s*, and iterated the removal process until all sequence coverages were within the bounds, amounting to 20 removed sequences.

The clock rate was estimated using TreeTime (28). We recorded the clock rate and standard error from the time tree constructed using the consensus sequences and compared this to the clock rate and standard deviations of the estimated clock rates in the resampled sequences. The tree built from consensus sequences had a clock rate of 6.5 × 10^−4^ with a standard error of 8.01 × 10^−5^. The mean of the clock rates for all of the sets of resampled sequences was 8.6 × 10^−4^ with standard deviation of 5.3 × 10^−4^, which is approximately 1.6 times as large as the standard error for the consensus sequences.

**Figure 5.**
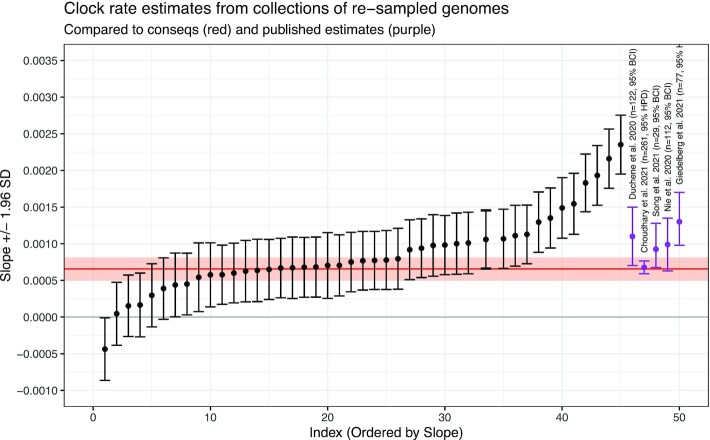
Clock rates (slope) and 95% confidence Intervals for the collections of re-sampled sequences. The red line and red shaded region are the clock rate and 95% CI for the consensus sequences. The purple points and error bars are the clock rates and error intervals (either Bayesian Credible Interval or Highest Posterior Probability) from published studies, as labelled. The re-sampled sequences are in line with the consensus sequences as well as the published sequences, but represent a much larger variation due to the uncertainty in the original genome sequences.

The estimates of the clock rate are shown in Figure 5. The red line and shaded region are the clock rate for the tree built from consensus sequences along with ±1.96 standard errors. Rate estimates from (30) (*n* = 122), (31) (*n* = 261), (32) (*n* = 29), (33) (*n* = 112) and (34) (*n* = 77) are also labelled on the plot with purple error bars for 95% Bayesian Credible Intervals (BCI) or 95% Highest Posterior Density (HPD), indicating that the rates and errors from each root-to-tip regression are in line with other published results. Figure 5 demonstrates that the estimated evolutionary rates have an average close to the rate estimated from our tree estimated from consensus sequences as well as the rates from other studies, but each of the individual error bars (from the five studies identified above) miss the excess variation due to sequence uncertainty.

## DISCUSSION

The primary contribution of this research is the construction of the probability sequence, which allows for a wide variety of future research directions. The direction we described here is focused on re-sampling, which allows a more complete appraisal of the variance in the estimates (or provides a reasonable prior distribution in a Bayesian setting), while comparing results for the most likely sequences provide a measure of robustness to sequence uncertainty.

Our proposed methods can result in a linear increase in computational expense. Even the method based on ordering the sequences by likelihood inevitably requires re-running the analysis numerous times. However, we have demonstrated that the uncertainty in the sequences themselves can lead to major changes to the interpretations of the results. The so-called ‘consensus sequence’ is simply the most likely sequence, and the reported uncertainty is not merely an academic curiosity. Ideally individual analyses would be constructed to take nucleotide-level uncertainty into account. For instance, phylogenies have been estimated based on uncertain sequence information in (35–37), but the uncertainty is not derived from base quality scores. An extension of these methods to incorporate the base quality scores is a worthwhile research direction.

As noted by a reviewer, De Maio *et al.* (38) present a method to construct phylogenetic trees such that each tip is associated with a collection of individuals within a species. It uses a multiple sequence alignment for each of a collection of species and incorporates the polymorphisms for each species. Our method could re-purpose this paradigm to apply to re-samples from the probabilistic sequence in place of multiple sequence alignments, with the separate genomes acting as species. Alternatively, the method could be altered to directly incorporate sequence uncertainty, possibly using values from our construction of the probabilistic sequence as allele proportions. This combination of methods would improve the estimation of the variance and allow for an improved estimate of error rate (analagous to the within-species evolution rate).

Computational burden can also be reduced by sorting the sequences in decreasing uncertainty. It is possible to devise an algorithm that puts the sequences in (approximate) order of their uncertainty without calculating the uncertainty for every sequence (specifically, by starting with the consensus and at each step changing the base call that had the lowest quality). Any model that uses sequence data could be re-fit with each sequence in order of uncertainty to investigate the robustness of that model to sequence uncertainty.

Our analysis focused on lineage classification according to the Pangolin model as well as estimation of the clock rate. The importance of incorporating sequence uncertainty is not confined to these applications; any analysis involving sequenced genomes would benefit from some method of incorporating the uncertainty or including some measure of robustness. For example, the estimated frequency of alleles in the population could be used as the probability sequence, then propagated into further analyses. We also included a section on assumptions about errors that are not quantified (consensus-level FASTQ and FASTA files), but we have not implemented an example of this. Evaluating particular methods was not part of our scope, but such a study would be a valuable research direction.

Within SARS-CoV-2, there are many potential use-cases for our methods. As noted by a reviewer, one potential use-case is to use simulated reads (with known lineage) with varying levels of uncertainty in order to estimate the potential variance around a given lineage assignment. It is likely that, due to different amounts of mutations used to define lineages and differences in average read depth at different locations, different lineages may be subject to different levels of variability. We stress that re-sampling is a general method, and development of methods that incorporate uncertainty—e.g. incorporating uncertainty directly in the inference procedure, perhaps directly in the formulation of the likelihood—should be a priority for future research in particular applications of uncertainty propagation.

Our method does not preclude tertiary analyses to test for systematic errors. For instance, in a post on virological.org (https://virological.org/t/issues-with-sars-cov-2-sequencing-data/473), Nicola De Maio *et al.* suggest that some errors arise due to issues in the sequencing protocol in particular laboratories. Our method allows for adjustments of the base call quality score, such as in (39), correcting for laboratory-specific errors, as well as more sophisticated definitions of genome likelihoods (e.g. (9,10,14)).

We have evaluated an algorithm to include insertion events in a re-sampling scheme, but many of the resultant sequences were not mappable to known sequences. The Pangolin lineage assignment system appears to treat insertions differently from single nucleotide polymorphisms, and our method of sampling insertions is incompatible with their treatment of them. This is potentially because the sampled base pair at any given position is independent of each other position, and the insertions observed in real-world data are possibly always associated with particular mutations elsewhere. However, insertions in the SARS-CoV-2 genome have been relatively rare.

This study should not be taken in any way as a criticism of the Pangolin lineage assignment procedure. Rather, Pangolin was chosen as it is the state-of-the art tool for lineage classification. The phylogeny created by this team has been a vital resource for researchers and for public health professionals. In particular, the PANGO label for the current Variants of Concern (VOCs), especially B.1.1.7, are the labels being used worldwide by news organizations. The output from Pangolin and many other bioinformatics tools are usually interpreted as *deterministic* results. This study is an argument that inherent uncertainty in sequencing warrants propagation into downstream analyses.

## CONCLUSIONS

The files produced by NGS platforms include valuable information about the quality of base calls which should be propagated into analyses. In this study, we have demonstrated that these errors in base calling can lead to different conclusions when determining a lineage via Pangolin and that the variance in clock rate estimates is larger than previously shown due to these errors. Both of these situations could lead to incorrect conclusions, such as missing a variant of interest or making overconfident conclusions about the date of the first case of COVID-19. The potential for errors in base calls should always be taken into account when making decisions based on genetic sequencing data.

Our analysis of Pangolin lineage classification demonstrates that the uncertainty in the base calls has a non-trivial2 effect on the potential lineage calls. The reported lineage classifications are based on a sophisticated classification algorithm which has high confidence in the predicted category, but this assumes that the input sequence is known without error. We are not aware of any classification system that incorporates per-base error, so we suggest that interpretations of the output of any classification system be interpreted with reference to the uncertainty in their sequence.

Our clock rate estimation suggest that the confidence/credible intervals for the published clock rates are underestimated. As with lineage classification, we are not aware of any clock rate estimation procedures that incorporate the uncertainty in the base calls of the sequences. Researchers should be conscious of this potential source of currently unacknowledged error when reporting any results from sequenced genomes.

## DATA AVAILABILITY

All data for this work have been previously published. Unique SRA identifiers are provided in the supplementary materials.

## Supplementary Material

lqad038_Supplemental_File
