# Supplementary material for: SUP: a probabilistic framework to propagate genome sequence uncertainty, with applications
Source: NAR Genom Bioinform. 2023 Apr 24;5(2):lqad038. doi: 10.1093/nargab/lqad038 (PMC10124968; doi:10.1093/nargab/lqad038)
Supplement: lqad038_Supplemental_File [file lqad038_supplemental_file.pdf]

Supplementary Material for SUP: A Probabilistic Framework to Propagate Genome Sequence Uncertainty, with Applications

David Champredon, Devan Becker, Connor Chato, Gopi Gugan, Art Poon

1 Accession Numbers

The following table lists all of the NCBI SRA accession numbers used in this paper.

ERR4085809	ERR4890354	ERR4892048	ERR5064166	ERR5082590	SRR13021020
ERR4204823	ERR4890371	ERR4892066	ERR5064294	ERR5082598	SRR13021022
ERR4305816	ERR4890386	ERR4892112	ERR5064346	ERR5082599	SRR13021027
ERR4307842	ERR4890403	ERR4892152	ERR5064787	ERR5082600	SRR13021032
ERR4440194	ERR4890427	ERR4892200	ERR5064811	ERR5082606	SRR13021033
ERR4440219	ERR4890531	ERR4892203	ERR5074314	ERR5082610	SRR13021035
ERR4440247	ERR4890572	ERR4892293	ERR5076163	ERR5082622	SRR13021038
ERR4440332	ERR4890609	ERR4892339	ERR5076748	ERR5082630	SRR13021042
ERR4440354	ERR4890693	ERR4892386	ERR5077151	ERR5082645	SRR13021047
ERR4440373	ERR4890746	ERR4892392	ERR5077411	ERR5082654	SRR13021052
ERR4440402	ERR4890771	ERR4892423	ERR5077618	ERR5082656	SRR13021053
ERR4440425	ERR4890819	ERR4893013	ERR5077713	ERR5082673	SRR13021059
ERR4440731	ERR4890820	ERR4893031	ERR5077924	ERR5082674	SRR13021061
ERR4692420	ERR4890881	ERR4893033	ERR5078210	ERR5082694	SRR13021067
ERR4692568	ERR4890926	ERR4893037	ERR5078863	ERR5082695	SRR13021072
ERR4692877	ERR4890974	ERR4893080	ERR5078897	ERR5082696	SRR13021073
ERR4692945	ERR4891001	ERR4893138	ERR5079000	ERR5082700	SRR13021077
ERR4693014	ERR4891011	ERR4893184	ERR5079423	ERR5082702	SRR13021084
ERR4693019	ERR4891037	ERR4893186	ERR5079699	ERR5082706	SRR13021090
ERR4693495	ERR4891061	ERR4893197	ERR5080131	ERR5082708	SRR13021093
ERR4693801	ERR4891103	ERR4893242	ERR5080159	ERR5082710	SRR13021097
ERR4693865	ERR4891178	ERR4893353	ERR5080327	ERR5082711	SRR13021098
ERR4694010	ERR4891235	ERR4893393	ERR5080504	ERR5082712	SRR13021099
ERR4694330	ERR4891238	ERR4999251	ERR5080893	SRR11433882	SRR13021104
ERR4694380	ERR4891261	ERR4999255	ERR5080897	SRR11433888	SRR13021107
ERR4694400	ERR4891304	ERR4999275	ERR5080913	SRR11433893	SRR13021109
ERR4694498	ERR4891415	ERR4999282	ERR5080918	SRR12639958	SRR13021111
ERR4694556	ERR4891433	ERR5060778	ERR5081077	SRR12639961	SRR13021113
ERR4694571	ERR4891444	ERR5062004	ERR5081293	SRR12749715	SRR13021115
ERR4694617	ERR4891493	ERR5062062	ERR5081301	SRR12749716	SRR13021124
ERR4759453	ERR4891497	ERR5062388	ERR5081304	SRR12762573	SRR13021130
ERR4869446	ERR4891532	ERR5062514	ERR5081316	SRR13020989	SRR13021131
ERR4869458	ERR4891572	ERR5062571	ERR5081322	SRR13020990	SRR13021133
ERR4869480	ERR4891675	ERR5062648	ERR5081836	SRR13020991	SRR13021134
ERR4869487	ERR4891711	ERR5062729	ERR5082214	SRR13020998	SRR13021135
ERR4869497	ERR4891715	ERR5062935	ERR5082346	SRR13020999	SRR13021143
ERR4890228	ERR4891805	ERR5063143	ERR5082556	SRR13021003	SRR13092002
ERR4890271	ERR4891841	ERR5063165	ERR5082561	SRR13021008	SRR13592146
ERR4890285	ERR4891863	ERR5063539	ERR5082569	SRR13021010	
ERR4890294	ERR4891889	ERR5063807	ERR5082576	SRR13021011	
ERR4890337	ERR4891898	ERR5063813	ERR5082578	SRR13021013	
ERR4890352	ERR4891988	ERR5063922	ERR5082580	SRR13021017	

Supplementary Table 1: Accession numbers for the resampling application. The prefix ERR indicates that the sequence comes from the European Nucleotide Archive, whereas the prefix SRR indicates that it comes from the NCBI's Short Read Archive.

ERR4333012	ERR4598849	ERR4645575	ERR4763502	ERR4893972	ERR5189526
ERR4422411	ERR4599609	ERR4647168	ERR4763917	ERR4905630	ERR5196271
ERR4423341	ERR4632053	ERR4651160	ERR4788047	ERR4906265	ERR5240413
ERR4423907	ERR4633143	ERR4652052	ERR4792808	ERR4989718	ERR5277731
ERR4424340	ERR4633439	ERR4652877	ERR4793403	ERR5020396	ERR5293387
ERR4424995	ERR4633631	ERR4659368	ERR4824581	ERR5021500	ERR5304264
ERR4437121	ERR4637060	ERR4667806	ERR4824816	ERR5025790	ERR5307708
ERR4437452	ERR4639632	ERR4668406	ERR4824949	ERR5027044	ERR5314268
ERR4438147	ERR4639682	ERR4668440	ERR4825023	ERR5040813	ERR5316846
ERR4438486	ERR4639875	ERR4668990	ERR4826433	ERR5041080	ERR5334211
ERR4459703	ERR4640333	ERR4669218	ERR4827255	ERR5052912	ERR5339018
ERR4459988	ERR4640467	ERR4686528	ERR4835054	ERR5052951	ERR5339175
ERR4460366	ERR4640673	ERR4686632	ERR4848811	ERR5054027	ERR5349715
ERR4463279	ERR4641513	ERR4686760	ERR4849464	ERR5057371	ERR5353067
ERR4581201	ERR4641559	ERR4688435	ERR4860936	ERR5058153	ERR5379217
ERR4584371	ERR4642761	ERR4688535	ERR4861339	ERR5177079	SRR12349113
ERR4584814	ERR4643065	ERR4699831	ERR4874605	ERR5181042	SRR12349131
ERR4597698	ERR4643184	ERR4706873	ERR4874858	ERR5187190	
ERR4597906	ERR4644945	ERR4763252	ERR4878261	ERR5188799	

Supplementary Table 2: Accession numbers used in the root-to-tip application. The prefix ERR indicates that the sequence comes from the European Nucleotide Archive, whereas the prefix SRR indicates that it comes from the NCBI's Short Read Archive.
